# A verification protocol for the probe sequences of Affymetrix genome arrays reveals high probe accuracy for studies in mouse, human and rat

**DOI:** 10.1186/1471-2105-8-132

**Published:** 2007-04-20

**Authors:** Rudi Alberts, Peter Terpstra, Menno Hardonk, Leonid V Bystrykh, Gerald de Haan, Rainer Breitling, Jan-Peter Nap, Ritsert C Jansen

**Affiliations:** 1 Groningen Bioinformatics Centre, Groningen Biomolecular Sciences and Biotechnology Institute, University of Groningen, 9751 NN Haren, The Netherlands; 2 Groningen Bioinformatics Centre, University Medical Centre Groningen, University of Groningen, 9713 AV Groningen, The Netherlands; 3 Department of Cell Biology, section Stem Cell Biology, University Medical Centre Groningen, University of Groningen, 9713 AV Groningen, The Netherlands; 4 Bioinformatics Expertise Center, Institute for Life Science & Technology, Hanze University Groningen, 9747 AS Groningen, The Netherlands

## Abstract

**Background:**

The Affymetrix GeneChip technology uses multiple probes per gene to measure its expression level. Individual probe signals can vary widely, which hampers proper interpretation. This variation can be caused by probes that do not properly match their target gene or that match multiple genes. To determine the accuracy of Affymetrix arrays, we developed an extensive verification protocol, for mouse arrays incorporating the NCBI RefSeq, NCBI UniGene Unique, NIA Mouse Gene Index, and UCSC mouse genome databases.

**Results:**

Applying this protocol to Affymetrix Mouse Genome arrays (the earlier U74Av2 and the newer 430 2.0 array), the number of sequence-verified probes with perfect matches was no less than 85% and 95%, respectively; and for 74% and 85% of the probe sets all probes were sequence verified. The latter percentages increased to 80% and 94% after discarding one or two unverifiable probes per probe set, and even further to 84% and 97% when, in addition, allowing for one or two mismatches between probe and target gene. Similar results were obtained for other mouse arrays, as well as for human and rat arrays. Based on these data, refined chip definition files for all arrays are provided online. Researchers can choose the version appropriate for their study to (re)analyze expression data.

**Conclusion:**

The accuracy of Affymetrix probe sequences is higher than previously reported, particularly on newer arrays. Yet, refined probe set definitions have clear effects on the detection of differentially expressed genes. We demonstrate that the interpretation of the results of Affymetrix arrays is improved when the new chip definition files are used.

## Background

Microarrays are widely used to study genome-wide gene expression levels. A frequently used type of microarray is the Affymetrix GeneChip [1]. This technology uses multiple probes per gene (probe set) to measure the amount of mRNA present (target). For reasons of specificity, probes are chosen to be complementary to a unique part of the target sequence. Although all probes from a single probe set should measure the same amount of mRNA, the hybridization signals of individual probes for a given mRNA molecule may vary widely. This is believed to be caused by variations in molecular characteristics of the probe sequence, such as GC content and secondary structure, and corrections have been proposed to calculate true expression levels averaged over probe signals [2,3]. However, another reason for the variation in signal between probes could be misdesigned probes, that either do not match the target RNA or can hybridize with other, non-target, RNA molecules. For correct interpretation of the results of Affymetrix GeneChip hybridizations, it is important to know which probes may cause variation in hybridization and for what reason. For example, in our large scale genetical genomics applications [4-6], individual probe hybridizations are used to map regulatory regions in a genome. In such applications, it is important to be able to rule out potential false positive results due to misdesigned probes.

An earlier analysis of the probe sequences of the Affymetrix mouse genome U74Av2 array [7] against the RefSeq database showed that for only 51% of the probe sets on the array all probes could be 'entirely verified', that is, corresponded without any mismatch to a RefSeq mRNA sequence. A recent analysis at the individual probe level verified 73% of the individual probe sequences of the MG-U74Av2 array against mRNA sequences from Entrez [8]. Affymetrix supplies regular updates of probe set verifications using new releases of the RefSeq, GenBank and Ensembl databases [9,10]. In the July 2006 release, 70% of the probe sets of the MG-U74Av2 GeneChip are 'entirely verified'. These surprisingly low verification percentages suggest that a major part of the hybridization results of such an array should be regarded with caution. Little information is available on the possibility of hybridization of individual mouse probes with non-target RNA molecules [8]. Here we present an extensive and generalized protocol for the verification of probe sequences on Affymetrix arrays.

The protocol uses four databases: NCBI RefSeq, NCBI UniGene Unique, NIA Mouse Gene Index, and UCSC mouse genome. By incorporating these databases in the verification protocol, the number of sequence-verified probes of the Affymetrix mouse arrays increases considerably. The same protocol applied to other mouse arrays, or a similar protocol (based on RefSeq, UniGene Unique and UCSC genome) for human and rat arrays, yielded similar results. Refined chip definition files (CDF files), which include only verified probes, are provided online.

We conclude that with the corrections as proposed previously [2,3], the accuracy and reliability of the Affymetrix arrays is considerably higher than reported till now. Our new data on probe verification and cross-hybridization are important for assessing unexpected behaviour of any given individual probe in a given experiment and will contribute to the more accurate assessment of expression data using Affymetrix arrays.

## Results

### Quality of sequence databases

The verification protocol for mouse arrays makes use of three messenger databases (NCBI RefSeq, NCBI UniGene Unique, NIA Mouse Gene Index), and one genome database (UCSC mouse genome). We first assessed their quality. Assuming that the genome is the most accurate sequence with an error rate of less than 1 in 10,000 bases [11,12], we compared the sequence of 1000 randomly selected genes, all occurring in each of the three messenger databases, to the genome (see Methods). Table 1 shows that there were no major quality differences between the messenger databases, except that the NIA Mouse Gene Index showed a lower mismatch frequency. The sequence differences that are observed may be due to sequencing errors, but also to genetic polymorphisms between mouse strains. This means that each database contains reliable information and can be used to verify probe sequences.

**Table 1:** Comparison of sequence databases

Database	No. of mismatches	No. of gaps	No. of nucleotides
RefSeq	2026	690	2291664
UniGene Unique	2076	717	2286979
NIA	337	703	1915516

Comparison with the genomic sequence of all entries of 1000 randomly selected genes that each has an entry in each of the three messenger databases. The summed number of mismatches, gaps and nucleotides for these 1000 genes is given.

### The verification protocol

In the protocol for mouse arrays, we use the BLAST program to verify all probe sequences against the three messenger databases (see Methods). Using the terminology of Mecham et al. [7], for each probe set we determine per database whether it is

• 'entirely verified', meaning that all probes were identical to a messenger sequence;

• 'partially verified', meaning that only a subset of probes was identical to a messenger sequence;

• 'entirely unverified', meaning that none of the probes was identical to a messenger sequence.

Only probe sets that could not be classified as 'entirely verified' against one of the three messenger databases, were verified against the genome (see Methods). Each probe set is assigned a verification score which is the best score over all databases, where 'entirely verified' is better than 'partially verified', and 'partially verified' is better than 'entirely unverified'. For the final verification score the order of the databases does not matter since each probe set is assigned the best possible score.

We included all mentioned databases in the protocol to obtain the greatest coverage. Since the genome sequence database is much larger than the messenger databases and therefore the verification against the genome takes much longer, we have put the verification against the genome in the last position. This improves computational efficiency. The verification is not hampered by the lower accuracy of the messenger databases compared to the genome, since only 0.60% (0.34%) of the probe sets of the MG-U74Av2 (430 2.0) array were 'entirely verified' against one of the messenger databases but 'entirely unverified' against the genome. We examined some of the probe sets that were 'entirely unverified' against the genome in more detail. These seem to represent contaminated non-mouse sequences, or the tiny fraction of genes that are still missing from the assembled genomes. Because there are no major quality differences between the messenger databases, their order is in principle arbitrary. However, we have put RefSeq in the first position in the protocol since it contains the most intensively curated transcript sequence information and probe sets that are 'entirely verified' against this database exit the protocol with RefSeq gene identifiers (supplementary material).

### Verification of the U74 and 430 arrays

**Figure 1:**
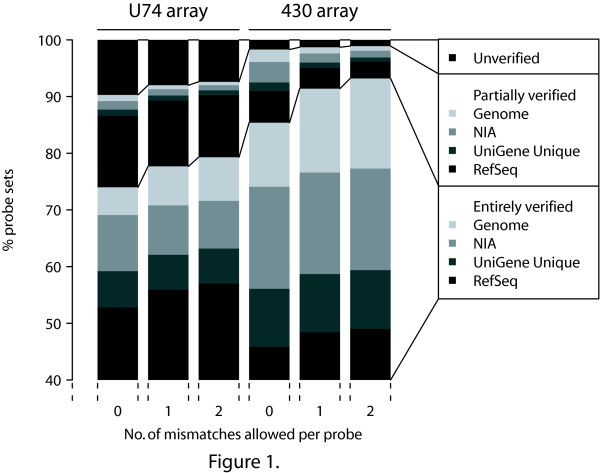
**Results of the verification protocol for the U74 and 430 arrays**. Three analyses were done per array: allowing only perfect matches, allowing one mismatch per probe and allowing two mismatches per probe. Per analysis, probe sets are assigned to the highest quality group (the lowermost group in the figure). So if a probe set is 'entirely verified' in RefSeq, it is assigned to this group. If it is not 'entirely verified' in RefSeq but it is 'entirely verified' in UniGene Unique, it is assigned to this second group, and so on.

We here report the results of the application of the verification protocol to two mouse GeneChips, MG-U74Av2 and 430 2.0, to which we will refer as 'U74' and '430', respectively. The percentages of 'entirely verified' probe sets are reported in a cumulative way, i.e. they grow with every database added; see Figure 1.

Megablasting all probe sequences of the U74 array against the mouse NCBI RefSeq database 'entirely verified' only 53% of all probe sets; this confirms the 51% reported earlier [7] with an older version of the RefSeq database. From the 430 array, only 46% of all probe sets could be 'entirely verified' (Figure 1). Next, by including the UniGene Unique database we 'entirely verified' 59% and 56% of all probe sets in U74 and 430, respectively. Then, by including the NIA Mouse Gene Index, the percentages grow to 69% and 74%, respectively. At last, we verified the remaining probe sets that were not yet 'entirely verified' against the UCSC mouse genome database. This way, we finally 'entirely verified' 74% and 85% of all probe sets in U74 and 430, respectively. More detailed numbers of the contribution of each of the databases to the final verification are given in additional file 1: 'Verification scores for the Affymetrix U74 array' and additional file 2: 'Verification scores for the Affymetrix 430 array'.

### Most 'partially verified' probe sets contain at most two bad probes

**Figure 2:**
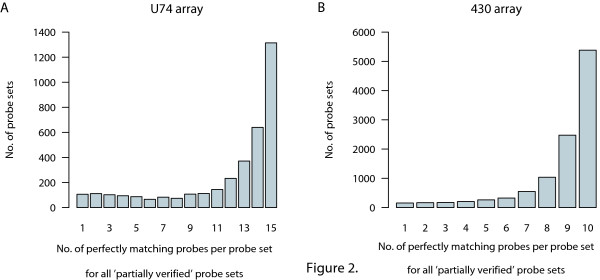
**Number of perfectly matching probes per probe set that are 'partially verified' against the genome**. (A) for 3644 probe sets of the U74 array; (B) for 10729 probe sets of the 430 array.

In this verification protocol, the class 'partially verified' is heterogeneous in nature. For a proper interpretation of the hybridization signals of a given probe set, it may be required to know how many and which probes of a particular set are not giving a perfect match with the mouse genome data available. In Figure 2 we have plotted the number of perfectly matching probes for those probe sets that were categorized in the 'partially verified' class. This shows that mainly one or two probes per probe set give a less than perfect match. Especially in case of the U74 array, where 16 probes per gene are present, the hybridization results of such non-perfect probes could be disregarded and the remaining probe set can be considered 'entirely verified'. In the supplementary material, the precise identification of these probes can be retrieved. By repeating the protocol and allowing one or two non-perfect probes per probe set, 80% and 94% of the probe sets of U74 and 430 were 'entirely verified', respectively.

### Allowing mismatches

Laboratory experience has shown that often the hybridization conditions do not allow distinction between a perfect match and a mismatch probe [13]. In this context, it could be argued that the requirement for a perfect match in probe sequence verification is not necessary, especially when only PM signals are used for estimating the expression levels, as is the case for most modern probe summarization methods (RMA, GCRMA). Moreover, messenger databases contain sequencing errors. For these two reasons, we have repeated the verification protocol as established above while allowing either one or two mismatches per probe sequence; 26% and 47% of the unverified probes had one or two mismatches between probe and target for U74 and 430, respectively. Figure 1 shows that the percentage of 'entirely verified' probe sets increases considerably, up to 77% for U74 and 91% for 430 in case of one mismatch and up to 79% for U74 and 93% for 430 for two mismatches. If we restrict ourselves to probe sets labeled by Affymetrix with "_at" then 85% of the probe sets are 'entirely verified' for U74 and 92% for 430 in case of one mismatch, and 87% for U74 and 93% for 430 for two mismatches. If we allow for two mismatches and also drop one or two unverifiable probes then 84% and 97% of all probe sets of U74 and 430 were 'entirely verified'. The hybridization conditions of the individual laboratory will have to decide which validation scheme is most appropriate and which probes or probe sets have to be scrutinized with more care.

### Cross-hybridization

**Figure 3:**
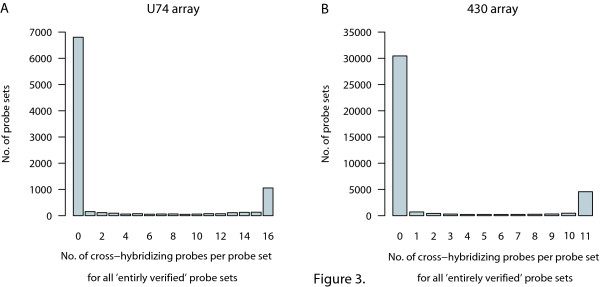
**Number of cross-hybridizing probes per probe set for the 'entirely verified' probe sets**. (A) for 9184 probe sets of the U74 array; (B) for 38476 probe sets of the 430 array.

Another issue of quality control is the specificity of probe sequences. A probe set may be 'entirely verified' with a given gene, yet an individual probe from such a set may be identical, or more similar than desired, to the sequence of another gene. This may cause cross-hybridization of different mRNAs and give rise to a probe that yields a hybridization signal that differs markedly from the other probe sequences. For the U74 array, 17% of the probes in 'entirely verified' probe sets had more than one Megablast hit against the RefSeq, UniGene Unique and/or NIA databases; 23% of the verified probe sets had at least one such probe with multiple Megablast hits. For the 430 array the percentages are 15% and 18% respectively. The numbers of cross-hybridizing probes per verified probe set are given in Figure 3. Note that the genome has not been used to assess cross-hybridization, since probe selection regions were used and individual probes were not compared with the genome. In the majority of probe sets with cross-hybridizing probes, all probes are cross-hybridizing. This indicates different splicing variants or duplicated genes that have different identifiers but can not be distinguished by these probe sets. Again, the individual laboratory will have to decide which probes or probe sets have to be scrutinized with more care.

### Verification of all available human, mouse and rat arrays confirms high probe accuracy

We applied the protocol to all other Affymetrix mouse arrays and we developed a similar protocol consisting of the three databases RefSeq, UniGene Unique and UCSC genome for the analysis of all human and rat Affymetrix arrays. Table 2 shows the results; the most striking observation is that, except the human X3P array, the newer arrays show high accuracy of probe sequences. New chip definition files for these 30 arrays can be downloaded from [14]. One can choose CDF files with or without cross-hybridizing probes and allowing for 0, 1 or 2 mismatches between sequence and probe.

**Table 2:** Percentage of verified probe sets for all Affymetrix human, mouse and rat arrays analyzed

array	(a)	(b)	(c)	(d)
HC-G110	80	91	97	Feb 19, 2002
HG-Focus	86	97	99	Jul 02, 2002
HG-U133A 2.0	84	96	98	Nov 07, 2003
HG-U133A	84	96	98	Feb 19, 2002
HG-U133B	80	96	98	Feb 19, 2002
HG-U133 Plus 2.0	82	96	98	Nov 07, 2003
HG-U95Av2	84	93	97	Feb 19, 2002
HG-U95B	78	92	97	Feb 19, 2002
HG-U95C	71	85	93	Feb 19, 2002
HG-U95D	68	83	93	Feb 19, 2002
HG-U95E	71	86	93	Feb 19, 2002
HuGeneFL	68	84	94	Feb 19, 2002
Human X3P	20	25	26	Jul 19, 2004
MG-U74Av2	74	81	84	Feb 19, 2002
MG-U74Bv2	71	83	87	Feb 19, 2002
MG-U74Cv2	39	49	61	Feb 19, 2002
MOE-430A	90	95	97	Jun 18, 2003
MOE-430B	81	92	96	Jun 18, 2003
Mouse 430 2.0	85	94	97	May 25, 2004
Mouse 430A 2.0	90	95	97	Jun 18, 2003
Mu11K-A	71	82	89	Feb 19, 2002
Mu11K-B	45	53	57	Feb 19, 2002
RAE-230A	80	94	97	Jun 19, 2003
RAE-230B	69	89	94	Jun 19, 2003
Rat 230 2.0	64	89	93	Jul 20, 2004
RG-U34A	60	69	74	Feb 19, 2002
RG-U34B	20	28	33	Feb 19, 2002
RG-U34C	22	32	38	Feb 19, 2002
RN-U34	81	87	91	Feb 19, 2002
RT-U34	66	73	79	Feb 19, 2002

(a) all probe sequences are sequence verified; (b) all probes, except at most two per probe set, are sequence verified; (c) all probes, except at most two per probe set, are sequence verified allowing for one or two mismatches between probe and target gene; (d) date of release of the array.

### The impact of updated probe set definitions on expression data

Microarrays are often used to find genes that are differentially expressed. To assess the impact of the updated probe set definitions on the assessment of differential gene expression, we reanalyzed an example dataset, the Clinical Prostate Cancer Behavior dataset (see Methods), consisting of 52 prostate tumor RNA samples and 50 non-diseased RNA samples hybridized to the human HG-U95Av2 array. Using RankProducts (Methods), we calculated lists of differentially expressed genes, both using the original Affymetrix CDF file and the new CDF file. 943 upregulated probe sets were detected with both CDF files, 32 probe sets were detected only with the new CDF file and 41 probe sets were detected only with the original CDF file (at a significance level of p < 0.05, Bonferroni adjused; similar numbers were found for the downregulated genes).

This result only shows that there are differences between the two CDF files. To check if refining the probe set definition indeed *improves *the results, we performed additional testing. For this purpose we focused on those genes that are most strongly affected, i.e. those genes whose rank in the list created with the original CDF file and the rank in the list created with the new CDF file are most different. Such genes will appear as differentially expressed in one list but not the other. If random probes are different between the two analyses, this difference can be both ways, with equal probability. However, we predict that improved probe sets will result in better detection of differential expression, as non-verifiable probes probably do not show differential expression and hence weaken the differential expression of the whole probe set. In that case, the genes that differ between the two lists should more often be detected as differentially expressed with the new CDF file compared to the original CDF file, than the other way around. Focusing on the genes with the highest differences in ranks, a significant proportion (p < 1E-10; Wilcoxon signed rank test) had a higher (better) rank in the list created with the new CDF file compared to the list created with the original CDF file (Table 3, columns a, b), confirming our prediction. Of the 250 probesets that showed the highest improvement in rank, 100% had been redefined. This indicates that a significant number of genes is picked up as differentially expressed with the new CDF file, while they remain undetected using the original CDF file.

**Table 3:** Comparison of lists of differentially expressed genes created with original and new CDF files.

	Prostate		Smokers		Male/female	
*n*	Up	Down	Up	Down	Up	Down

20	95%	100%	95%	100%	100%	100%
100	92%	97%	97%	99%	91%	98%
250	82%	94%	97%	92%	86%	95%
500	74%	86%	89%	84%	79%	86%
1000	55%	69%	78%	72%	75%	64%
2000	43%	48%	62%	52%	74%	54%

Two ranked lists of differentially expressed genes were calculated: one using Affymetrix' original CDF file and one using our CDF file. Genes were sorted in descending order according to their normalized difference in rank (|rank1–rank2|/minimum(rank1, rank2)). The top *n *genes are those which are most strongly affected by the probe set redefinition. The percentage of genes that obtained a higher rank using our refined CDF file compared to using the original CDF file is given. For random redefinitions, a percentage of 50 would be expected. Separate analyses for up- and downregulated genes were done. It can be seen that among the most strongly affected probe sets the large majority shows improved differential expression results when analyzed using the refined CDF file.

To verify that this observed improvement of results is consistent in other datasets and platforms, we repeated this evaluation procedure for a dataset of 34 smoker vs. 23 non-smoker samples from intra-pulmonary airway epithelial cells hybridized to HG-U133A arrays and a dataset of 4 male vs. 4 female BWF1 lupus-prone mice spleen samples hybridized to MG-U74Av2 arrays. We saw the same clear improvement, with high statistical significance (Table 3). As outlined above, we expect that random changes in the probe set definition would lead to equal numbers of genes being affected in either direction. We calculated the difference of the observed amount of genes having a higher rank with the new CDF and the expected amount (*n*/2), for different values of *n*. We used the maximum excess as an estimate of the number of probe sets that are significantly improved by refining the CDF files. Depending on the array, these numbers range between 321 and 658. Although these numbers are small compared to the total number of genes present on the array, they comprise a large fraction of the genes that are typically found to be differentially expressed in a microarray experiment.

## Discussion

In different studies [7,8,15,16] Affymetrix probe sequences were verified against mRNA databases. In all of these studies, only one mRNA database was considered. Gautier et al. [15] and Zhang et al. [16] verified human Affymetrix arrays against mRNA sequences from Entrez and RefSeq. Elo et al. [8] investigated the reproducibility of the probe signals for different generations of Affymetrix arrays. They compared the correlations of probe signals for original Affymetrix probe sets and verified probe sets, which they defined as the subset of probes of the original probe sets that only match with the target transcript for which the probes were originally designed by Affymetrix. They found that probe verification improved the correlations between generations of Affymetrix arrays and also that probe verification improved the consistency of the measurements within an array. Mecham et al. [7] showed that probe verification results in increased precision in technical replicates; increased accuracy across complementary microarray platforms, increased accuracy translating data from oligonucleotide arrays to cDNA microarrays, and increased diagnostic power of microarray technology.

A problem with the RefSeq and the UniGene Unique databases is that 3' UTRs are often truncated by the way the sequences are assembled [17,18], while Affymetrix selects the probes from the 600 bases most proximal to the 3' end of each transcript [19]. We overcame this problem by incorporating the genome in the verification protocol, where all 3' UTRs are available.

The Fantom 3 project (Functional annotation of the mouse, [20]) provides an extensive characterization of the mouse transcriptome. We also tested the verification protocol with the Fantom 3 transcripts included. Since this did not increase our verification scores (data not shown), we did not include this database in our protocol.

The mRNA and genome databases currently available are mainly based on the C57BL/6 mouse strain. Also, the probes on the Affymetrix arrays are mainly based on the C57BL/6 mouse strain. When samples from C57BL/6 mice are hybridized to the arrays, their transcripts are expected to perfectly match the probes. However, mice from genetically different strains or from recombinant inbred pedigrees, as in our genetical genomics applications [4,6], may carry allelic SNPs compared to the C57BL/6 genome. Probes carrying allelic SNPs may hamper data interpretation as putative differential mRNA expression can be confounded with differential hybridization [4]. When sequences of other mouse strains become available, the verification protocol here developed should be repeated for these newly sequenced strains to identify and, if so desired, eliminate probes carrying allelic SNPs.

The use of refined probe set definitions, that exclude unverified probes, will improve the interpretation of expression data, as non-hybridizing and mis-hybridizing probes add only noise to the data. Our evaluation of expression data from the public domain shows that this is indeed the case.

## Conclusion

By combining various verifications as described above, we show that 74% of the U74 probe sets and 85% of the 430 probe sets can be considered 'entirely verified' when based on perfect matches. When two mismatches are allowed, the percentages increase to 79% for U74 and 93% for 430. When considering individual probes, 85% and 95% of the probes were verified for U74 and 430 respectively, and even 89% and 97% when allowing two mismatches. Our extensive analyses of probe sequence data show that the inclusion of various databases, such as the genome sequence, indicate that the arrays are much more accurate than shown previously. Existing data can be reanalyzed with our verified probe sets (using the online CDF files). We show that such a refined probe set definition has clear effects on the detection of differentially expressed genes and demonstrate for various experiments that the results are systematically improved by discarding unverified probes.

## Methods

### Affymetrix GeneChips

Probe set annotations and probe selection regions (PSR) for all human, mouse and rat arrays, were obtained from Affymetrix [9,10].

The U74Av2 array is based on the mouse UniGene database, release 74. It contains 196.670 oligomers of length 25, divided into 12.422 probe sets, most of which contain 16 oligomers. Probe sets of the newer 430 2.0 array were selected from sequences derived from dbEST (NCBI, June 2002), GenBank (NCBI, Release 129, April 2002), and RefSeq (NCBI, June 2002) [21]. It contains 495.374 oligomers of length 25, divided into 45.037 probe sets, generally consisting of 11 oligomers.

### Sequence databases

RefSeq is a curated non-redundant collection of naturally occurring DNA, RNA and protein sequences. It is based on the sequences and annotations supplied to GenBank by the original researchers [17]. For mouse we used 55,810 messenger sequences from RefSeq.

UniGene is a processed and curated collection of millions of ESTs (Expressed Sequence Tags), which are relatively inaccurate (around 2% error). To assign ESTs to genes, the ESTs are clustered and the cluster consensus sequences stored in UniGene Unique [18]. The mouse UniGene Unique release contains 43,104 sequences.

NIA Mouse Gene Index (developed by the National Institute on Aging) is currently the most comprehensive collection of alternative transcription/splicing sequences. Patterns of alternative transcription/splicing are obtained by aligning a complete and nonredundant transcriptome assembly from expressed sequences (obtained from RefSeq, GenBank, dbEST, Ensembl and NIA) to the mouse genome [22]. The NIA Mouse Gene Index contains 186,405 sequences.

The UCSC mouse genome (maintained by University of California Santa Cruz) reports about 90% of the genome in finished form (error rate of less than 1 in 10,000 bases). We used build mm7 (corresponding to NCBI build 35.1; August, 2005).

For the mouse protocol we used two NCBI [23] databases: RefSeq mRNAs (NCBI, Feb. 3, 2006) and UniGene Unique (NCBI, build 151, Oct. 20, 2005). In addition, we used all mouse mRNA sequences from the National Institute on Aging (NIA Mouse Gene Index 5, June 2005, [22]) and the UCSC mouse genome (mm7, Aug. 2005, [11]). For the human protocol we used RefSeq mRNAs (NCBI, Feb. 16, 2006), UniGene Unique (NCBI, build 188, Dec. 30, 2005) and UCSC human genome (hg17, May 2004). For the rat protocol we used RefSeq mRNAs (NCBI, Feb. 16, 2006), UniGene Unique (NCBI, build 149, Jan. 25, 2006) and UCSC rat genome (rn3, June 2003).

### Assessment of the quality of the sequence databases

To assess the quality of the sequence databases, we took the UCSC genome sequence as a reference, and compared the sequences of 1000 randomly selected genes, all occurring in each of the three messenger databases, to the genome sequence. Since the genome contains introns and the messenger databases do not, we extracted the exon sequences from the genome by using the exon coordinates of RefSeq genes and attached them to each other. Then for each of the 1000 genes we compared the three messenger sequences to the reconstructed genome sequence and counted the amounts of mismatches and gaps (Table 1).

### Sequence alignment algorithms

Individual probes were analyzed against the messenger databases with Megablast (version 2.2.6 with a word size of 12, [24]) for 'short nearly exact matches'. Hits in databases were distinguished on the basis of none, one or two mismatches with the probe sequence.

Since analysis of all single probe sequences against the mouse genome gives too many non-exon hits (data not shown), we used the probe selection region (PSR) of each probe set as input for BLAT ([11], standalone BLAT version 32 × 1, standard settings). PSR is defined as the unique part of the messenger sequence from which Affymetrix selected the probes [19]. We masked all nucleotides not represented in probe sequences. Within the obtained BLAT hits of the masked PSRs, we re-identified the position of each probe to count the number of mismatches per probe.

### Computing infrastructure

All analyses were performed on a Linux cluster consisting of 200 nodes with dual Opteron processors 2 GHz and 1 GB memory. The average computation time per array was 4 hours on one node.

### Datasets and methods for determining the impact of updated probe set definitions on expression data

The Clinical Prostate Cancer Behavior dataset was downloaded from [25]. The smoker vs. non-smoker dataset was downloaded from the Gene Expression Omnibus (GEO) and has accession number GSE994. The male vs. female BWF1 lupus-prone mice dataset was also downloaded from GEO (accession number GSE2336). In all cases we used RMA [26] to generate probe set-level data. Using RankProducts [27] we calculated ranked lists of differentially expressed genes using Affymetrix' original CDF file and our refined CDF file, while separating up- and downregulated genes.

## Authors' contributions

RA was responsible for designing and implementing the protocol, interpreting the data, writing and finalizing the paper. PT helped in the design of the protocol, supervised the implementation and contributed to the interpretation of the data and writing the paper. MH participated in the implementation of the protocol. LVB helped with the biological interpretation of the results. GdH participated in writing the paper. RB participated in determining the impact of updated chip definition files on expression data and finalization of the manuscript. JPN drafted the manuscript, helped with the interpretation of the data and contributed to the finalization of the manuscript. RCJ conceived the study of individual Affymetrix probes, coordinated the study and the writing and was responsible for the funding. All authors read and approved the final manuscript.

## Supplementary Material

Additional File 1 Verification scores for the Affymetrix U74 array. The file contains the numbers and percentages of probe sets per occurring combination of verification scores. 'e' = 'entirely verified', 'p' = 'partially verified' and 'u' = 'entirely unverified'. Click here for file

Additional File 2 Verification scores for the Affymetrix 430 array. The file contains the numbers and percentages of probe sets per occurring combination of verification scores. 'e' = 'entirely verified', 'p' = 'partially verified' and 'u' = 'entirely unverified'. Click here for file
